# Phosphorus Acquisition Efficiency Related to Root Traits: Is Mycorrhizal Symbiosis a Key Factor to Wheat and Barley Cropping?

**DOI:** 10.3389/fpls.2018.00752

**Published:** 2018-06-05

**Authors:** Pedro Campos, Fernando Borie, Pablo Cornejo, Juan A. López-Ráez, Álvaro López-García, Alex Seguel

**Affiliations:** ^1^Scientific and Technological Bioresource Nucleus BIOREN-UFRO, Universidad de La Frontera, Temuco, Chile; ^2^Department of Soil Microbiology and Symbiotic Systems, Estación Experimental del Zaidín-Consejo Superior de Investigaciones Científicas, Granada, Spain; ^3^Departamento de Ciencias Químicas y Recursos Naturales, Universidad de La Frontera, Temuco, Chile; ^4^Section Ecology and Evolution, Biological Institute, University of Copenhagen, Copenhagen, Denmark

**Keywords:** cereal, phosphorus, fungal diversity, mycorrhizae, nutrient uptake, PAE, root traits

## Abstract

Wheat (*Triticum aestivum* L.) and barley (*Hordeum vulgare* L.) are major crops cultivated around the world, thus playing a crucial role on human diet. Remarkably, the growing human population requires a significant increase in agricultural production in order to feed everybody. In this context, phosphorus (P) management is a key factor as it is component of organic molecules such as nucleic acids, ATP and phospholipids, and it is the most abundant macronutrient in biomass after nitrogen (N), although being one of the scarcest elements in the lithosphere. In general, P fertilization has low efficiency, as only a fraction of the applied P is acquired by roots, leaving a substantial amount to be accumulated in soil as not readily available P. Breeding for P-efficient cultivars is a relatively low cost alternative and can be done through two mechanisms: i) improving P use efficiency (PUE), and/or ii) P acquisition efficiency (PAE). PUE is related to the internal allocation/mobilization of P, and is usually represented by the amount of P accumulated per biomass. PAE relies on roots ability to acquire P from the soil, and is commonly expressed as the relative difference of P acquired under low and high P availability conditions. In this review, plant adaptations related to improved PAE are described, with emphasis on arbuscular mycorrhizal (AM) symbiosis, which is generally accepted to enhance plant P acquisition. A state of the art (1980–2018) of AM growth responses and P uptake in wheat and barley is made to discuss about the commonly accepted growth promoting effect and P increased uptake by AM fungi and the contrasting evidence about the generally accepted lack of positive responses in both plant species. Finally, the mechanisms by which AM symbiosis can affect wheat and barley PAE are discussed, highlighting the importance of considering AM functional diversity on future studies and the necessity to improve PAE definition by considering the carbon trading between all the directly related PAE traits and its return to the host plant.

## Introduction

Cereals have been cultivated for more than 10,000 years, playing a crucial role in the development of human civilization. Today, cereals are still important, being the principal crops harvested in the world with more than 2.8 Gt of combined grain production (FAO, 2013). Among major cereals, the widespread and closely related wheat (*Triticum aestivum* L.) and barley (*Hordeum vulgare* L.) represents 31% of global grain yield (El Rabey et al., 2015). Cereals are also the major component of human diet worldwide with more than 50% of daily caloric intake, with values exceeding 80% in the poorest countries (Awika, 2011). Agricultural practices and technology have greatly improved over the last decades to reduce problems associated with food scarcity and to provide cereals for the daily diet. However, risks and unprecedented challenges still remain considering that global food, and grain production must increase a 70% by the year 2050 as world population is expected to be reach 9 billion people (FAO, 2012). Meanwhile, the slight increase in crop yields observed since the 1980s and the scarcity of available land suitable for production make the focus on reducing crop losses empirical due to various kinds of biotic and abiotic stresses factors, such as pathogen attack, cold, heat, drought, salt, deficiency of nutrients as phosphorous (P), and phytotoxicity by heavy metal stresses (Ray et al., 2012; Bhardwaj et al., 2014).

P fertilizers are manufactured from rock phosphate found only in a few places in the world, being Morocco the owner of 85% of the known active mining reserves. As a non-renewable resource, rock phosphate, as well as other non-renewable resources such as oil and coal is expected to become scarce near the 2030s (Cordell et al., 2009), or more optimistically within two to three centuries (Sattari et al., 2012). The market and countries are already responding to this scenario, which is reflected in the fact that both USA and China (the biggest P producer in the world) have stopped exporting this resource (van de Wiel et al., 2016). In addition, P fertilizers may cause environmental problems associated with eutrophication (Gaxiola et al., 2001) and can contain heavy metals such as cadmium that may accumulate in arable soils as a result of the addition of rock phosphate (van de Wiel et al., 2016). Remarkably, usually only about 10–30% of the P fertilizer applied in the first year is taken up by the roots, with a substantial part accumulated in the soil as residual P not readily available for plants (Syers et al., 2008). In alkaline soils, P can be bound to calcium, and in acidic soils it can be readily complexed to charged Al and Fe oxides and groups hydroxyls on clay surfaces (Kochian et al., 2004; Seguel et al., 2013), limitations that can occur in *ca*. 30% of arable soils worldwide (Kochian, 2012). Moreover, organic material present in the soil (e.g., from manure or crop residues) can also bind phosphate ions as well as phytate (inositol compounds).

Ideally, P taken up by agricultural products should represent the same amount of applied P fertilizer, achieving a neutral balance (Helyar, 1998; Syers et al., 2008). However, this situation is often only achieved in low input, low production farming systems (e.g., Burkitt et al., 2007; McIvor et al., 2011), on intrinsically low P-buffering capacity soils in productive agriculture (e.g., sands), or where P-buffering capacity is low because sorption sites for P are close to saturation and soil P availability is relatively high (e.g., Syers et al., 2008). Elsewhere, P-balance is relatively low, which contributes to an inefficient P use (Richardson et al., 2011). Thus, P management must be improved in order to enhance plant uptake in soils, as well as using the less available and bound-P through a better understanding of the processes happening in the soil-plant systems.

## Phosphorus in the soil-plant continuum

In general, P is present in plants either as organic phosphate esters or as free inorganic orthophosphate forms, representing up to *ca*. 0.2% of plants dry weight, making it the most abundant macronutrient in plants after nitrogen (N). However, unlike N, the amount of P available for agriculture is finite (Bovill et al., 2013). When compared to other essential macronutrients, P is one of the less-abundant elements in the lithosphere (0.1% of the total). P is an important component of organic molecules such as nucleic acids, ATP and phospholipids; thus playing a crucial role in energy metabolism of both plants and animals (Abel et al., 2002; Vance et al., 2003). Phosphate is also involved in signal-transduction pathways via phosphorylation/dephosphorylation, hence regulating key enzyme reactions in general cellular metabolism, including N fixation on N-fixing plants (Theodorou and Plaxton, 1996; Schachtman et al., 1998; Marschner, 2012).

**Figure 1:**
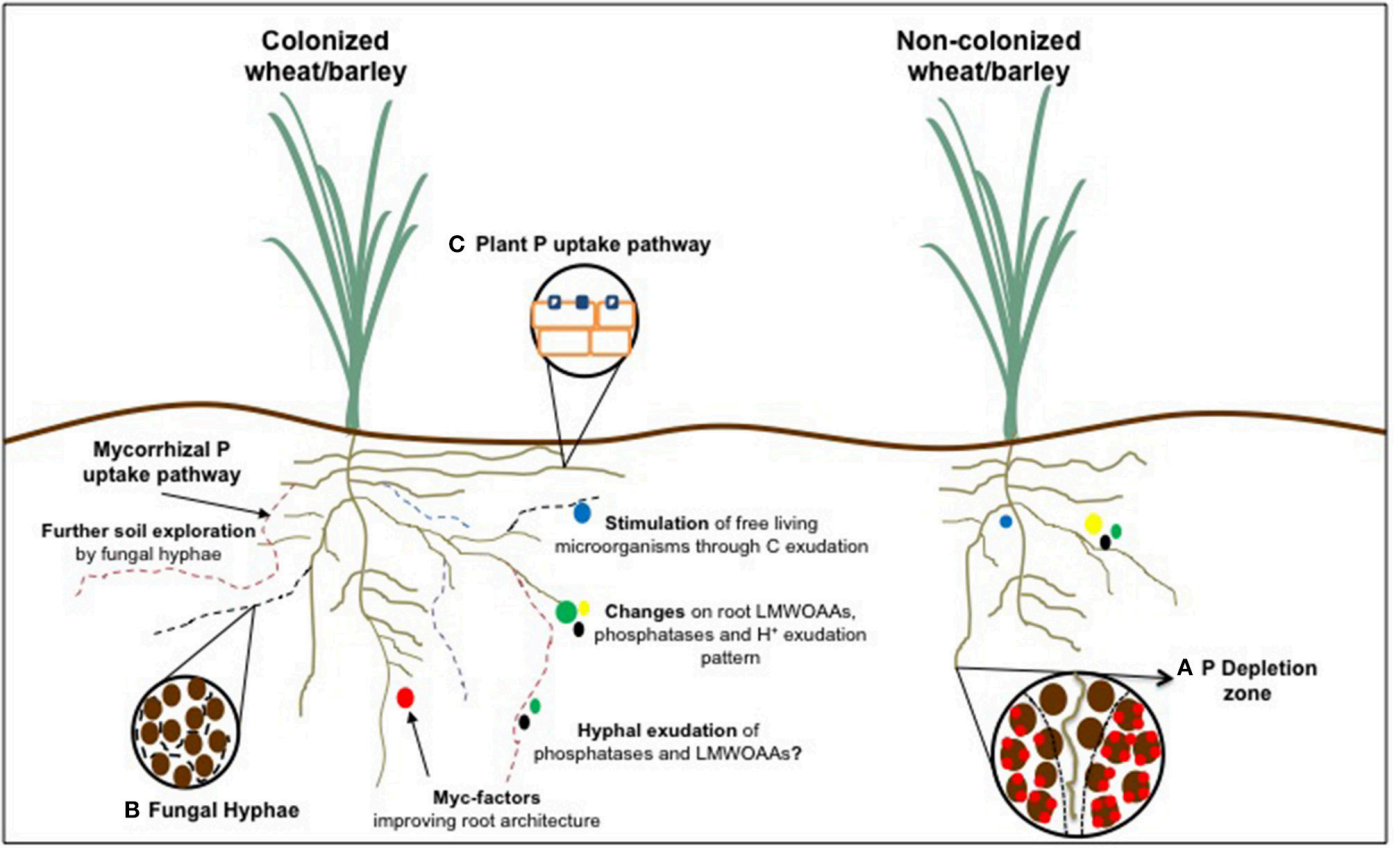
Phosphorus acquisition efficiency related traits of wheat and barley roots affected by arbuscular mycorrhizal symbiosis in comparison to a non-colonized counterpart. **(A)** Representation of P depletion zone around the rhizosphere; **(B)** Access to smaller soil pores by AM fungal hyphae; and **(C)** Modulation of plant P transporters following colonization.

Plants acquire P from the soil solution predominately as inorganic phosphate (Pi) (H_2_${\text{PO}}_{4}^{-}$/${\text{HPO}}_{4}^{-2}$), having maximal uptake rates at pH 5-6 (Holford, 1997; Rae et al., 2003; Marschner, 2012). It acquisition occurs by a plasma membrane-localized phosphate transporter-mediated process, which has been suggested to operate as a H^+^ co-transporter (Rae et al., 2003; Raghothama, 2005). Phosphate transporters are classified into four families: Pht1, Pht2, Pht3, and Pht4, which are located on the plasma membrane, plastidial membrane, mitochondrial membrane, and Golgi-compartment, respectively (Lopez-Arredondo et al., 2014). Two different uptake systems have been proposed: one with high-affinity, which is regulated by Pi availability and activated mainly under P deficiency, with a Km of 3–7 μM; and the other is a low-affinity system constitutively expressed system with Km of 50–300 μM (Bucher et al., 2001; Preuss et al., 2010; Liu et al., 2011; Tian et al., 2017). Despite of having a high-affinity acquisition system, P has a low availability and poor mobility in the soil, being one of the most inaccessible elements for plants (Holford, 1997). Concentrations of available P in soil solution are extremely low, being generally lower than 10 μM (Holford, 1997; do Nascimento et al., 2016), whereas in wheat leaves and stems concentrations of over 100 mM can be achieved (Bauer et al., 1987; Seguel et al., 2017). Therefore, as plants normally take up P faster than it is supplied by diffusion a depletion zone around the root system is quickly created, inducing P deprivation (Figure 1A; Hinsinger, 2001).

The rhizosphere encompasses the first millimeters of the soil surrounding plant roots, where biological and ecological complex processes occur. This is the critical zone for P dynamics as plants roots are capable of modifying this environment through their physiological activities, especially by exudation of organic acid anions, enzymes, secondary metabolites and sugars (Bais et al., 2006; Giles et al., 2017). These processes not only determine solubilization/mineralization, acquisition of soil nutrients and microbial dynamics, but also control the efficiency of nutrient use by plants and crops, therefore influencing productivity and sustainability of the agroecosystems (Hinsinger et al., 2009; Zhang et al., 2010).

## Phosphorus efficiency

Great efforts have been made in the last decade concerning P efficiency. In this sense, agronomic strategies for increasing P fertilizer availability to crops has been developed, for example, by applying liquid fertilizers (Holloway et al., 2001) or by localized fertilizer placement (Ma et al., 2009). However, those techniques require modern technologies and increase operational costs. On the other hand, breeding for P-efficient crop cultivars has been advocated due to its relatively low cost, providing benefits to both high and low-input systems (Rose et al., 2010).

Despite the growing knowledge in the field, there is still controversy in the concept and measurement of efficiency, as it has many definitions, and even different terms are often used although they are calculated in the same way (Bovill et al., 2013). Nowadays, P efficiency is understood as two different mechanisms: i) the internal efficiency of allocation/mobilization of P in order to produce higher biomass with lower input, and ii) plant ability to acquire P from the soil, also known as P acquisition efficiency (Wang et al., 2010; Rose and Wissuwa, 2012; Sandaña and Pinochet, 2016).

The internal use efficiency or P use efficiency (PUE) is here defined as the amount of P accumulated in the tissue per biomass unit (shoot and/or root) or grain produced (Rose and Wissuwa, 2012). It is related to a range of metabolic modifications that can occur for reducing P demand during plant development (Hammond et al., 2009; Vaneklaas et al., 2012). Improving internal PUE will lead to a more resource-efficient use of P rather than increasing uptake of potentially scarce P forms, as in theory less P will be acquired by crops, minimizing P fertilizer requirement and removal from fields. However, to date no crop species or genotypes within species are known to be capable of reducing its net P uptake if the demand is reduced (Rose and Wissuwa, 2012). This is operating in sandy or low P-soption capacity soils. On the contrary, in soils rich in sorbed P, which are observed in the majority of acid soils, breeding programs focused on the optimization of P scavenging mechanisms would be a key role to improve P efficiency. Consenquently, this review has been mainly focused on P acquisition efficiency.

### Phosphorus acquisition efficiency

**Figure 2:**
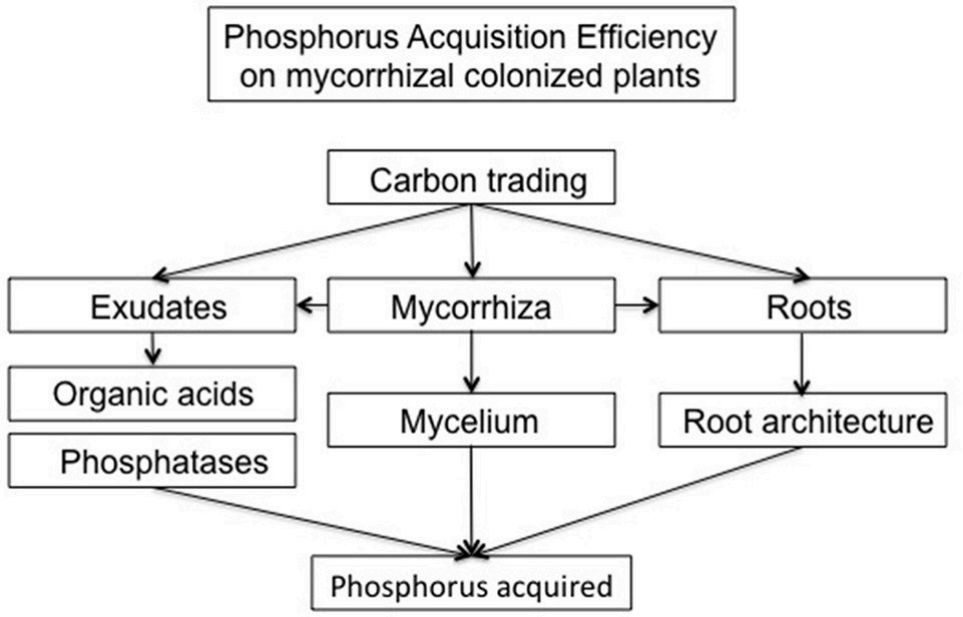
General scheme showing the proposed PAE determination based on carbon trading between all directly related P acquisition traits in AM colonized plants.

While PUE aims to produce more biomass with lesser P costs, P acquisition efficiency (PAE) is related to enhancing its acquisition from soil, especially from unavailable forms, and for this purpose root traits are a key factor. PAE is commonly expressed in the literature as the relative difference of P taken up in low and high P availability conditions (Vandamme et al., 2013; Seguel et al., 2015, 2017). However, this definition does not take into account the root traits involved. In this sense, Liao et al. (2008) made a more realistic approximation by integrating root length and root biomass. Nevertherless, other traits related to root architecture and physiolgy must be integrated in the PAE definition due to their key role in uptake as discussed below (Figure 2).

#### Root architecture

P status is a major factor modulating root architecture, being a higher root-to-shoot ratio the most evident change in the majority of plants experiencing P deprivation (Wissuwa et al., 2005; Gruber et al., 2013). Phosphate, the available form of P, presents a heterogeneous distribution (patches) given its high affinity for the soil matrix. Root P gathering implies a continuous root growth due to the quickly depletion of rhizosphere P and the need of looking for new hotspots in soils (Figure 1A).

The upper soil layer (0–10 cm)—known as topsoil—is the zone where P availability for plants and microorganisms is generally higher, mainly due fertilizers input in the surface and its poor mobility throught soil profile. Important adaptations of plants to access this richer environment are the development of axial roots with shallower angle, enhancing adventitious rooting, and greater density and dispersion of lateral roots and root hairs (Wang et al., 2004; Lynch, 2007). These traits, together with root length, diameter and surface area comprise the most important inter- and intra-specifically functional variations of plant root adaptations for PAE for most plant species. During their screening for traits directly related to PAE, Manske et al. (2000) found that higher root length density in top soil of wheat crops was the most important root trait for P uptake, which was positively correlated with enhanced recovery of fertilized P. Basal roots in some legumes (as bean and soybean) appear in distinct nodes or “whorls,” which affect root growth angles and therefore top soil exploration. Differences of up to 100% of improved P acquisition can be found in common bean cultivars as basal root whorl number varies among genotypes (Lynch, 2007; Miguel, 2011). However, a certain tradeoff occurs between P and water uptake since plants with higher density of roots in top soil and shallower angles have lower water use efficiency, as water is usually more abundant in deeper layers under drought conditions (Ho et al., 2005). Another obstacle in improving root density is the associated carbon cost of producing root hairs, that have to be compensated by producing either smaller or thinner hairs and/or increased proportion of aerenchyma in the cortex and less secondary growth of the stele (Lynch and Ho, 2005; Zhu et al., 2010). Plants would otherwise spare carbon allocated in developing “productive” parts.

Modeling root traits are clearly advantageous strategy for enhancing PAE. However, screening and phenotyping for these traits remain a complex challenge as soil-based study systems are high technology based, and hydroponic/aeroponic systems cannot totally emulate the complexity of the processes occurring in the soil. Therefore, genotypes selected in this way do not always show their superiority in field trials (van de Wiel et al., 2016).

#### Root exudates

If P is present on fixed sources and/or unavailable forms, plants having larger and/or more branched root architecture do not significantly improve P acquisition. In this case, root physiology and biochemical responses play a major role on accessing P from sparingly available pools in soil. Hence, the exudation of low molecular weight organic acids (LMWOAAs), proton extrusion, phosphatase exudation and/or association with symbiotic and non-symbiotic microorganisms present in the rhizosphere are the most important adaptations developed by plants (Figure 1).

As inorganic P forms availability and enzymatic activity are strongly affected by soil pH (Hinsinger, 2001), P solubility can be increased by root-induced acidification in alkaline soils or by pH increase of the rhizosphere in acidic and deeply weathered soils (Gahoonia et al., 1992; Jones and Oburger, 2011). This process occurs mainly because changes in pH in the rhizosphere can influence surface charges on soil particles and therefore Pi availability (Geelhoed et al., 1999). Plants have the ability to either increase or decrease rhizospheric pH up to 2–3 pH units, mainly by absorption or release of protons in order to equilibrate cation/ anion balance (Hinsinger et al., 2003). In the specific case of the cereals wheat and barley, Gahoonia and Nielsen (1996) observed that when rhizospheric pH was invariable, the plants displayed significant genotypic variation in terms of PAE, indicating that other mechanisms should also be involved in causing variation on P acquisition.

Carboxylates and the corresponding carboxylic acids, also known as LMWOAAs, constitute the major fraction of root exudates during P deficiency (Figure 1). Usually, the most common organic acid anions found in rhizosphere are lactate, acetate, oxalate, succinate, fumarate, malate, citrate, isocitrate, and aconitate (Jones, 1998). They have distinct functions on energetic cell metabolism, maintaining charge balance or osmotic potential. It has been widely suggested that LMWOAAs can improve P availability by mobilizing sparingly available P forms in the soil solution. This occurs by chelating metals ions like Al, Fe or Ca involved in P sorption and occupying sorption sites on minerals (Jones, 1998). P mobilizing activity through LMWOAAs is based on their variable negative charge, which would allow the complexation of metal cations and the displacement of anions from the soil matrix. The above is supported by several studies reporting an increase of organic acids exudation by roots in response to P deprivation, especially in plants from Proteacea family that possess cluster roots (Jones, 1998; Vance et al., 2003; Delgado et al., 2013). In addition, the presence of LMWOAAs in solution has been seen to increased P availability compared to water treatments (Gerke, 1992; Khademi et al., 2009, 2010). The efficiency in mobilizing P differs across LMWOAAs as follows: citrate > oxalate > malate > acetate. However, organic acid anion-induced P release depends on many factors, such as pH, soil mineralogy and anion concentration (>100 mM for citrate, >1 mM for oxalate, malate and tartrate) (Bolan et al., 1994; Jones and Darrah, 1994; Lan et al., 1995). Indeed, the rates to which Pi and organic anions are replaced in soil solution make predictions of the real effect difficult. Organic acid anions have a fast turnover as they can be quickly adsorbed in acidic soils and rapidly degraded in alkaline counterparts, with half-lives of several hours (Wang et al., 2010). Contrasting evidence found that, despite exuding citrate, pea genotypes were not capable of mobilizing P from Al-P and Fe-P complexes (Pearse et al., 2007). Nevertheless, organic acid production constitutes an important carbon cost in plant metabolism, with 5–25% of total fixed carbon by photosynthesis being used to sustain exudation. However, this does not seem to significantly affect net biomass production as P deficiency can reduce growth to an even greater extent (Johnson et al., 1996; Keerthisinghe et al., 1998).

Sparingly available organic P forms represent between 30% and 90% of total P in some soils (Borie et al., 1989; Jones and Oburger, 2011). Substantial flows of P occur between inorganic and organic P pools in soil through immobilization and mineralization, being both processes mediated predominantly by soil microorganisms (Oberson and Joner, 2005; Richardson and Simpson, 2011). In order to utilize this P source, organic compounds have to be mineralized; that is, organic P substrates must be hydrolyzed by enzymatic activity of phosphatases to release Pi. This activity seems to be more pronounced in the rhizosphere and it is associated with a depletion of soil organic P (Gahoonia and Nielsen, 1992; Chen et al., 2002; Spohn and Kuzyakov, 2013). Phosphatases are enzymes responsible for catalyzing the hydrolysis of phosphoric acid anhydrides and esters (Schmidt and Laskowski, 1961). These are classified by the Nomenclature Committee of the International Union of Biochemistry and Molecular Biology into 5 groups: phosphomonoesterases (EC 3.1.3), phosphodiesterases (EC 3.1.4), triphosphoric monoester hydrolases (EC 3.1.5), enzymes acting on phosphoryl-containing anhydrides (EC 3.6.1) and on P–N bonds (EC 3.9) (Nannipieri et al., 2011). Phosphomonoesterases are the most abundant enzymes in soils and include acid and alkaline forms and phytases, among others. To date, there is no evidence that any plants produce alkaline phosphomonoesterases.

There is an increasing interest on phytases due to the fact that they hydrolyze inositol phosphates (isomers and lower order derivatives of inositol hexakisphosphate) which generally constitute a major component of soil total organic P. Ranging from 4 to 40% of total P in soils (Borie et al., 1989; Smernik and Dougherty, 2007; Turner, 2007), inositol phosphates are readily adsorbed to soil particles and can react with cations (Fe and Al in acidic soils and Ca in alkaline ones) depending on pH to form poorly soluble precipitates (Shang et al., 1992; Celi and Barberis, 2005). However, in most plant species phytase activity has limited capability to mineralize inositol phosphate due to its low production and exudation from roots and the poor availability of the substrate in solution (Richardson et al., 2001; George et al., 2007). Attempts to creating transgenic plants overexpressing phytases and/or other phosphatases have been achieved (Lung et al., 2005; Wasaki et al., 2009) with little successes under natural soil conditions, where substrate availability is restricted (Lung and Lim, 2006; Wang et al., 2009). Interestingly, phosphatase activities are higher near the rhizosphere, with maximum activities found from 2 to 3.1 mm to the root surface for acid and 1.2 to 1.6 mm for alkaline phosphomonoesterases, showing a negative correlation with rhizospheric organic P content in wheat plants (Nannipieri et al., 2011). Phosphatase activity is also regulated by other factors, such as soil mineralogy, organic matter content, P availability and bacterial communities present in the rhizosphere (Joner and Jakobsen, 1995; Snajdr et al., 2008; Stursova and Baldrian, 2011).

#### Microorganisms

Non-symbiotic soil microorganisms play a key role on organic P ecosystem dynamics (Figure 1; Harvey et al., 2009; Khan et al., 2010). It has been proposed that all alkaline phosphomonoesterases found in soil have a microbial origin, mainly bacterial (Tabatabai, 1994; Yadav and Tarafdar, 2003). Additionally, the majority of Pi mineralized from phytase activity is mediated by free-living bacteria and fungi (Unno et al., 2005; Richardson and Simpson, 2011). Spohn et al. (2013) using the ^33^P isotopic approach found that the release of root exudates could be a plant strategy to increase P mineralization by enhancing microbial activity.

Free-living soil microorganisms are believed to be more efficient than plants in absorbing and incorporating P into their biomass. Therefore, microbial P represents an important soil sink (Xu et al., 2013) and a potential source of available P for most plants as microbial P is located in more labile intracellular compounds with a fast turnover (Oberson and Joner, 2005; Bünemann et al., 2013; Hinsinger et al., 2015). Despite having an important role in organic P dynamics, most research related to free-living soil microorganisms to enhance PAE has been focused on microorganisms capable of solubilizing sparingly available P (Wakelin et al., 2004; Leggett et al., 2007). Microorganisms can release protons, LMWOAAs, and other secondary organic metabolites that may contribute to P solubilization from minerals (Jones and Oburger, 2011). Indeed, between 1-50% of soil bacteria and about 0.5-0.1% of soil fungi can be classified as P-solubilizing microorganisms (Kucey et al., 1989; Gyaneshwar et al., 2002). Fungal isolates (particularly the Genus *Penicillium*) have been largely studied due to their great capacity for solubilizing Pi in both solid and liquid media (Gyaneshwar et al., 2002; Leggett et al., 2007; Morales et al., 2011). A group of bacteria, usually denominated as plant-growth-promoting rhizobacteria (PGPR), are widely found in the rhizosphere of cropping and wild species and have the potential of enhancing PAE mainly through influencing nutrient availability, such as P, or via the indirect production of phytohormones, or plant growth regulators (Richardson et al., 2009). Among the latter, classical phytohormones as auxin, cytokinin, ethylene, gibberellin, and abscisic acid are included. These regulators influence root architecture and other features related to plant development (Peleg and Blumwald, 2011; Vacheron et al., 2013). Although the benefits of using PAE enhancing microorganisms have been evidenced in laboratory and glasshouse conditions, inconsistent results have been observed in field trials (Goos et al., 1994; Karamanos et al., 2010), with the exception of arbuscular mycorrhizal symbiosis established with certain soil fungi.

## Arbuscular mycorrhizal symbiosis

Mycorrhizal symbiosis is an association between plant and some fungal species that generally colonize root or rhizoids, and is beneficial to both partners, at least under some circumstances (Jansa et al., 2011). Arbuscular mycorrhizal (AM) is the most common and widespread type of mycorrhizal symbiosis (Trappe, 1987; Wang and Qiu, 2006; Smith and Read, 2008), found in *ca*. 80% of plant species among all major plants lineages (Wang and Qiu, 2006; Brundrett, 2009) and in most of agricultural species (exceptions include *Brassica* spp., and *Lupinus* spp.). Although AM symbiosis is facultative for many plant species, fossil evidence indicates that the symbiosis matches with the first appearance of land plants, more than 400 million years ago, playing a crucial role in the development of terrestrial plants (Bonfante and Genre, 2008; Brundrett, 2009). AM fungi (subphylum Glomeromycotina) are obligate biotrophs that when associated with plant roots can provide an enhanced foraging system in order to improve acquisition of soil water and nutrients, particularly P, and to improve resistance to biotic and abiotic stresses in exchange of energy (using carbohydrates as trade) for fungal growth and reproduction (Jansa et al., 2003a; Smith and Read, 2008; Jung et al., 2012; Pozo et al., 2015; Armada et al., 2016; Santander et al., 2017). P appears to be one of major regulators of AM symbiosis establishment and efficiency, as root colonization, P uptake through fungal pathway (Figure 1) (See section Changes in P Transporters) and growth responses diminish with increasing soil P availability (Smith and Read, 2008; Richardson et al., 2011; Smith et al., 2011). However, plants can also modulate the symbiosis, by stimulating fungal metabolic activity and hyphal branching among other effects (Bücking and Shachar-Hill, 2005; Besserer et al., 2006), through the exudation of strigolactones (Akiyama et al., 2005; Parniske, 2006; López-Ráez et al., 2017) Accordingly, the production of these strigolactones is promoted by P deprivation, although in wheat can be also promoted in a small fraction by N deficiency (Yoneyama et al., 2007, 2012; López-Ráez et al., 2008).

Despite its broad host range and that its cosmopolitan distribution, AM diversity involves only ~250 morphologically and 350 to 1000 molecularly defined AM fungi (Kivlin et al., 2011; Öpik et al., 2014), with low endemism patterns at global scale (Davison et al., 2015). The absence of AM fungal colonization is rare in natural conditions in plants able to perform the symbiosis, only being achieved in soils lacking AM fungal propagules or in non-mycorrhizal (NM) plant species (Smith et al., 2011). Commonly, the difference in plant growth in presence and absence of mycorrhizal fungal partners is defined as mycorrhizal growth responses (MGR) and vary widely from positive to negative depending on plant/fungi species and growth conditions (Johnson et al., 1997; Klironomos, 2003). When compared to MGR observed from other cereal crops [positives responses in maize (Sylvia et al., 1993; Karasawa et al., 2001) and rye (Baon et al., 1994a); and excluding rice, which is often not colonized or poorly colonized under continuous submersion (Vallino et al., 2009)], wheat and barley plants present a high variable response to AM colonization, being generally considered as low and sometimes showing even negative effects in plant growth (Hetrick et al., 1996; Grace et al., 2009). However, positive responses can also be found when applying different experimental conditions or analyzing at different growth stages, which indicates that AM fungal inoculation under appropiated circumstances can be an effective agronomic practice also in these crops (Borie and Rubio, 1999; Seguel et al., 2016a,b, 2017).

Interestingly, a recent meta-analysis by Pellegrino et al. (2015) looking at wheat responses to AM symbiosis inoculation under field conditions found out that although straw biomass was weakly correlated with root AM fungal colonization rate, grain yield and P accumulation correlated positively. A review of the main mycorrhizal growth responses and P uptake from mycorrhizal and NM treatments in wheat (Table 1A) and barley (Table 1B) are presented below highlighting the idea that growth responses associated to AM symbiosis are not directly related to P acquisition. Growth depletions upon AM fungal colonization are normally attributed to an excess of photosynthates shared with the fungal partner, which are estimated to be up to 20% of the C fixed by the host plant (Jakobsen, 1995; Ortas et al., 2002; Li et al., 2005; Morgan et al., 2005). However, some studies indicated that growth depletion resulting from C drain to the fungal symbiont do not apply in all cases. Hetrick et al. (1992) and Grace et al. (2009) reported that growth reductions in wheat and barley did not vary when associated with two different AM fungal partners with contrasting capacity to colonize their roots (e.g., 61 and 5%, respectively), and therefore, hypothetically different C demand from the host plant (Hetrick et al., 1992). Even so, cereals benefits from AM symbiosis despite growth and/or nutritional benefits (such as net P uptake) are not apparent. Special techniques such as isotopic labeling are necessary to demonstrate symbiosis functioning (nutrient, water and carbohydrate exchange) in these cases (Smith et al., 2004, 2009; Grace et al., 2009).

**Table 1A:** Mycorrhizal growth responses (MGR) and P uptake on mycorrhizal (+AM) and non-colonized (–AM) wheat (*T. aestivum* L.) cultivars under greenhouse or field conditions and at different days after sowing (DAS).

**Wheat cultivar**	**AM specie**	**MGR (%)**	**P uptake (mg/g)**		**Exp. conditions**	**Harvest (DAS)**	**Observation**	**References**
			**+AM**	**−AM**				
TAM-105	*G. etunicatum*	22	5.20	4.63	Field	175		Al-Karaki et al., 2004
Steardy	*G. etunicatum*	19	5.43	4.67	Field	175		Al-Karaki et al., 2004
Tam-105	*G. mossae*	6	4.73	4.63	Field	175		Al-Karaki et al., 2004
Steardy	*G. mossae*	6	5.20	4.67	Field	70		Al-Karaki et al., 2004
Tormes	*G. mossae*	36	1.6	1.2	Pot	70		Azcón and Ocampo, 1981
Anza	*G. mossae*	27	1.3	0.9	Pot	70		Azcón and Ocampo, 1981
Negrillo	*G. mossae*	−2	0.7	0.8	Pot	70		Azcón and Ocampo, 1981
7 Cerros	*G. mossae*	107	1.5	0.8	Pot	70		Azcón and Ocampo, 1981
Bastion	*G. mossae*	35	1.3	1.1	Pot	70		Azcón and Ocampo, 1981
Pane 247	*G. mossae*	15	2.0	1.3	Pot	70		Azcón and Ocampo, 1981
Lozano	*G. mossae*	28	1.9	1.6	Pot	70		Azcón and Ocampo, 1981
Cocorit	*G. mossae*	87	1.3	1.0	Pot	70		Azcón and Ocampo, 1981
Champlein	*G. mossae*	4	0.9	0.9	Pot	70		Azcón and Ocampo, 1981
Castan	*G. mossae*	3	1.9	1.8	Pot	70		Azcón and Ocampo, 1981
Tajo	*G. mossae*	4	1.8	1.6	Pot	70		Azcón and Ocampo, 1981
Boulmiche	*G. mossae*	3	1.1	1.0	Pot	70		Azcón and Ocampo, 1981
Jupateco	*G. mossae*	0	1.5	1.2	Pot	70		Azcón and Ocampo, 1981
Neepawa	*G. intraradices*	−27	1.5	1.1	Pot	42		Goh et al., 1997
Neepawa	*G. intraradices*	−29	2.6	2.9	Pot	42	50 mg P/kg	Goh et al., 1997
Neepawa	*G. intraradices*	−11	3.8	4.1	Pot	42	100 mg P/kg P	Goh et al., 1997
Neepawa	*G. intraradices*	−24	5.0	6.1	Pot	42	300 mg P/kg	Goh et al., 1997
Newton	*G. etunicatum + G. mosseae + G. intraradices*	−27	2.7	0.8	Pot	98		Hetrick et al., 1996
Turkey	*G. etunicatum + G. mosseae + G. intraradices*	160	1.4	0.8	Pot	98		Hetrick et al., 1996
Lewjain	*G. intraradices*	−7	1.56	1.33	Field	Tillering		Mohammad et al., 1998
Lewjain	*G. intraradices*	10	1.17	1.06	Field	Anthesis		Mohammad et al., 1998
Lewjain	*G. intraradices*	19	0.82	0.70	Field	Harvest		Mohammad et al., 1998
Lewjain	*G. intraradices*	5	1.76	1.80	Field	Tillering	30 kg P/ha	Mohammad et al., 1998
Lewjain	*G. intraradices*	−4	1.26	1.28	Field	Anthesis	30 kg P/ha	Mohammad et al., 1998
Lewjain	*G. intraradices*	11	0.93	0.71	Field	Harvest	30 kg P/ha	Mohammad et al., 1998
Diamondbird	*G. intraradices*	8	1.8	1.5	Field	122		Ryan and Angus, 2003
Diamondbird	*G. intraradices*	−9	2.3	2.3	Field	122	20 kg P/ha	Ryan and Angus, 2003
Diamondbird	*Scutellospora calospora*	8	2.1	1.5	Field	122		Ryan and Angus, 2003
Diamondbird	*Scutellospora calospora*	−5	2.1	2.3	Field	122	20 kg P/ha	Ryan and Angus, 2003
HPW-89	*G. mosseae* (local)	15	2.69	2.42	Field	150		Suri et al., 2011
HPW-89	*G. intraradices*	14	2.78	2.42	Field	150		Suri et al., 2011
HPW-89	*G. mosseae* (IARI)	13	2.79	2.42	Field	150		Suri et al., 2011
HPW-89	*G. mosseae* (local)	94	3.14	2.42	Field	150	50% P2O5 based on STCR	Suri et al., 2011
HPW-89	*G. intraradices*	103	3.36	2.42	Field	150	50% P2O5 based on STCR	Suri et al., 2011
HPW-89	*G. mosseae* (IARI)	95	3.34	2.42	Field	150	50% P2O5 based on STCR	Suri et al., 2011
HPW-89	*G. mosseae* (local)	154	3.67	2.42	Field	150	75% P2O5 based on STCR	Suri et al., 2011
HPW-89	*G. intraradices*	153	3.82	2.42	Field	150	75% P2O5 based on STCR	Suri et al., 2011
HPW-89	*G. mosseae* (IARI)	151	3.65	2.42	Field	150	75% P2O5 based on STCR	Suri et al., 2011
Laura	*G. clarum*	−10	1.42	1.10	Pot	95	0 mg P/kg	Xavier and Germida, 1997
Laura	*G. clarum*	−19	2.16	2.77	Pot	95	5 mg P/kg	Xavier and Germida, 1997
Laura	*G. clarum*	12	2.76	2.22	Pot	95	10 mg P/kg	Xavier and Germida, 1997
Laura	*G. clarum*	−7	2.43	2.67	Pot	95	20 mg P/kg	Xavier and Germida, 1997
Neepawa	*G. clarum*	17	0.42	0.57	Pot	95	0 mg P/kg	Xavier and Germida, 1997
Neepawa	*G. clarum*	−8	0.68	0.55	Pot	95	5 mg P/kg	Xavier and Germida, 1997
Neepawa	*G. clarum*	4	1.03	1.07	Pot	95	10 mg P/kg	Xavier and Germida, 1997
Neepawa	*G. clarum*	12	1.00	1.72	Pot	95	20 mg P/kg	Xavier and Germida, 1997
81(85)	*G. versiforme*	3	1.03	0.77	Pot	56		Yao et al., 2001
Fengxiao 8	*G. versiforme*	39	0.98	0.70	Pot	56		Yao et al., 2001
NC37	*G. versiforme*	21	1.06	0.91	Pot	56		Yao et al., 2001
HD 2204	*G. fasciculatum*	78	1.10	1.02	Field	135		Khan and Zaidi, 2007
HD 2204	*G. fasciculatum*	146	1.15	1.02	Field	135	A. chrococum	Khan and Zaidi, 2007
HD 2204	*G. fasciculatum*	155	1.89	1.02	Field	135	Bacillus	Khan and Zaidi, 2007
HD 2204	*G. fasciculatum*	295	1.76	1.02	Field	135	A. chrococum + Bacillus	Khan and Zaidi, 2007
HD 2204	*G. fasciculatum*	178	1.56	1.02	Field	135	A. chrococum + P. variable	Khan and Zaidi, 2007
HD 2204	*G. fasciculatum*	193	1.57	1.02	Field	135	A. chrococum + Bacillus + P. variable	Khan and Zaidi, 2007
WH 283	*Glomus* sp. 88	15	0.17	0.18	Pot	55		Singh and Kapoor, 1999
WH 283	*Glomus* sp. 88	42	0.20	0.18	Pot	55	B. circulans	Singh and Kapoor, 1999
WH 283	*Glomus* sp. 88	51	0.20	0.18	Pot	55	C. herbarum	Singh and Kapoor, 1999
WH 283	*Glomus* sp. 88	97	0.19	0.18	Pot	55	B. circulans + C. herbarum	Singh and Kapoor, 1999
Star	*G. mosseae*	17	2.5	2.2	Pot	60	Bavendorf soil, 200 mg P/kg	Tarafdar and Marschner, 1994b
Star	*G. mosseae*	16	1.4	0.8	Pot	60	Bavendorf soil, 200 mg organicP/kg	Tarafdar and Marschner, 1994b
Star	*G. mosseae*	28	2.3	2.0	Pot	60	Niger soil, 200 mg P/kg	Tarafdar and Marschner, 1994b
Star	*G. mosseae*	22	1.5	0.7	Pot	60	Niger soil, 200 mg organicP/kg	Tarafdar and Marschner, 1994b
UP 2003	*G. fasciculatum*	6	2.63	0.42	Pot	80		Zaidi and Khan, 2005
UP 2003	*G. fasciculatum*	136	1.0	0.42	Pot	80	A. chroococum	Zaidi and Khan, 2005
UP 2003	*G. fasciculatum*	142	1.61	0.42	Pot	80	P. striata	Zaidi and Khan, 2005
UP 2003	*G. fasciculatum*	236	1.10	0.42	Pot	80	A. chroococum + P. striata	Zaidi and Khan, 2005
UP 2003	*G. fasciculatum*	108	1.31	0.42	Pot	80	A. chroococum + P. variable	Zaidi and Khan, 2005
UP 2003	*G. fasciculatum*	122	1.5	0.42	Pot	80	A. chroococum + P. variable + P. striata	Zaidi and Khan, 2005

**Table 1B:** Mycorrhizal growth responses (MGR) and P uptake on mycorrhizal (+AM) and non-colonized (–AM) barley (*H. vulgare* L.) cultivars under greenhouse or field conditions and at different days after sowing (DAS).

**Barley cultivar**	**AM specie**	**MGR (%)**	**P uptake (mg/g)**		**Exp. conditions**	**Harvest (days)**	**Observation**	**References**
			**+AM**	**−AM**				
Vodka	*G. intraradices*	−4	0.28^*^	0.18^*^	Pot	80	0 mg P/kg	Plenchette and Morel, 1996
Vodka	*G. intraradices*	−12	0.29^*^	0.21^*^	Pot	80	20 mg P/kg	Plenchette and Morel, 1996
Vodka	*G. intraradices*	−8	0.32^*^	0.24^*^	Pot	80	30 mg P/kg	Plenchette and Morel, 1996
Vodka	*G. intraradices*	−7	0.37^*^	0.27^*^	Pot	80	40 mg P/kg	Plenchette and Morel, 1996
Vodka	*G. intraradices*	−11	0.48^*^	0.34^*^	Pot	80	50 mg P/kg	Plenchette and Morel, 1996
Vodka	*G. intraradices*	−6	0.45^*^	0.41^*^	Pot	80	60 mg P/kg	Plenchette and Morel, 1996
Vodka	*G. intraradices*	−8	0.44^*^	0.42^*^	Pot	80	70 mg P/kg	Plenchette and Morel, 1996
Vodka	*G. intraradices*	−7	1.06^*^	0.76^*^	Pot	80	110 mg P/kg	Plenchette and Morel, 1996
Vodka	*G. intraradices*	−3	1.65^*^	1.06^*^	Pot	80	160 mg P/kg	Plenchette and Morel, 1996
Vodka	*G. intraradices*	3	3.07^*^	2.92^*^	Pot	80	310 mg P/kg	Plenchette and Morel, 1996
cv. SLB-6	*G. mosseae*	14	2.33	1.29	Pot	45	120 spores/100g dry soil	Al-Karaki and Clark, 1999
cv. SLB-6	*G. mosseae*	39	2.77	1.29	Pot	45	240 spores/100g dry soil	Al-Karaki and Clark, 1999
cv. SLB-6	*G. mosseae*	27	2.17	1.29	Pot	45	360 spores/100g dry soil	Al-Karaki and Clark, 1999
Pallas P02	*G. claroideum + G. intraradices*	−17	1.32	1.32	Pot	28		Jakobsen et al., 2005
brb	*G. claroideum + G. intraradices*	46	1.75	1.45	Pot	28	root hairless mutant	Jakobsen et al., 2005
UC 566	*G. constrictus*	49	0.83	0.88	Pot	80		Jensen, 1982
UC 566	*G. fasciculatus* n.185	38	0.97	0.88	Pot	80		Jensen, 1982
UC 566	*G. fasciculatus* n. 0–1	45	1.00	0.88	Pot	80		Jensen, 1982
UC 566	*Gigaspora margarita*	−14	0.73	0.88	Pot	80		Jensen, 1982
Rupal	*G. fasciculatus* no. 0–1	2	2.82	2.79	Pot	102		Jensen, 1984
Rupal	*G. fasciculatus* no. 92	6	3.20	2.79	Pot	102		Jensen, 1984
Rupal	*G. epigaeus*	19	3.02	2.79	Pot	102		Jensen, 1984
Rupal	*Gigaspora margarita*	1	2.62	2.79	Pot	102		Jensen, 1984
Rupal	*G. mosseae CA*	0	2.98	2.79	Pot	102		Jensen, 1984
Rupal	*G. mosseae DK*	5	3.07	2.79	Pot	102		Jensen, 1984
Rupal	*G. mosseae GB*	7	3.23	2.79	Pot	102		Jensen, 1984
Rupal	*G. caledonius*	3	3.43	2.79	Pot	102		Jensen, 1984
Rupal	*G. macrocarpus* CA	13	3.12	2.79	Pot	102		Jensen, 1984
Rupal	*G. macrocarpus* DK	6	3.36	2.79	Pot	102		Jensen, 1984
Rupal	*G. etunicatus*	4	3.74	2.79	Pot	102		Jensen, 1984
Lofa Abed	*G. mosseae*	0	4.38	4.35	Pot	23	No sterilized	Khaliq and Sanders, 1998
Lofa Abed	*G. mosseae*	0	2.26	2.46	Pot	52	No sterilized	Khaliq and Sanders, 1998
Lofa Abed	*G. mosseae*	−14	2.07	1.95	Pot	67	No sterilized	Khaliq and Sanders, 1998
Lofa Abed	*G. mosseae*	−15	1.96	1.81	Pot	91	No sterilized	Khaliq and Sanders, 1998
Lofa Abed	*G. mosseae*	−13	2.17	2.08	Pot	116	No sterilized	Khaliq and Sanders, 1998
Lofa Abed	*G. mosseae*	−5	4.72	5.21	Pot	23	Sterilized	Khaliq and Sanders, 1998
Lofa Abed	*G. mosseae*	−20	2.29	2.59	Pot	52	Sterilized	Khaliq and Sanders, 1998
Lofa Abed	*G. mosseae*	−24	2.57	2.04	Pot	67	Sterilized	Khaliq and Sanders, 1998
Lofa Abed	*G. mosseae*	−23	2.33	1.64	Pot	91	Sterilized	Khaliq and Sanders, 1998
Lofa Abed	*G. mosseae*	−26	2.3	1.57	Pot	116	Sterilized	Khaliq and Sanders, 1998
Lofa Abed	*G. mosseae*	−3	0.17	0.16	Field	124	Sterilized 0 kg P/ha	Khaliq and Sanders, 2000
Lofa Abed	*G. mosseae*	−2	0.2	0.18	Field	124	Sterilized 100 kg P/ha	Khaliq and Sanders, 2000
Lofa Abed	*G. mosseae*	−2	0.13	0.12	Field	124	No sterilized 0 kg P/ha	Khaliq and Sanders, 2000
Lofa Abed	*G. mosseae*	−2	0.14	0.14	Field	124	No sterilized 100 kg P/ha	Khaliq and Sanders, 2000
ACSAD 6	Mix	37	2.27	1.97	Pot	35	Soil A	Mohammad et al., 2003
ACSAD 6	Mix	87	2.54	1.97	Pot	35	Soil A + 25 mg P/kg	Mohammad et al., 2003
ACSAD 6	*G. intraradices*	40	2.07	1.97	Pot	35	Soil A	Mohammad et al., 2003
ACSAD 6	Mix	28	2.76	2.29	Pot	35	Soil B	Mohammad et al., 2003
ACSAD 6	Mix	4	2.69	2.29	Pot	35	Soil B + 25 mg P/kg	Mohammad et al., 2003
ACSAD 6	*G. intraradices*	14	2.42	2.29	Pot	35	Soil B	Mohammad et al., 2003
ACSAD 6	Mix	22	2.63	1.80	Pot	35	Soil C	Mohammad et al., 2003
ACSAD 6	Mix	20	2.78	1.80	Pot	35	Soil C + 25 mg P/kg	Mohammad et al., 2003
ACSAD 6	*G. intraradices*	5	2.22	1.80	Pot	35	Soil C	Mohammad et al., 2003
Galleon	*G. intraradices*	−15	1.96	1.98	Pot	48	Soil temperature 10°C	Baon et al., 1994b
Galleon	*G. intraradices*	−26	2.45	2.3	Pot	48	Soil temperature 15°C	Baon et al., 1994b
Galleon	*G. intraradices*	−5	2.39	2.19	Pot	48	Soil temperature 20°C	Baon et al., 1994b

* *Phosphorus concentration on grain*.

### Mycorrhizal influence on PAE traits of wheat and barley

#### Root architecture and surface area

The root systems of grain cereals as wheat and barley consist of two types of roots. The first type is known as primary or seminal roots, and comprises between three to seven roots growing from the seedling. They have 0.2–0.4 mm diameter, occupying 5–10% of total root volume in mature plants. The second type is the secondary roots, also called nodal, crown, or adventitious roots. These roots emerge from nodes at the base of main stem and tillers 1–3 months after germination, having a larger diameter (0.3–0.7 mm) than primary roots (Hoad et al., 2001). Significant genetic variation for root architectural traits has been found among cereal cultivars (Kujira et al., 1994; Marschener, 1998). Interestingly, it was found out that the number of tillers positively correlated with root length density and grain yield of semidwarf bread wheat cultivars grown under P deficiency (Manske et al., 2000). In addition, Gahoonia et al. (1997) showed that the presence of root hairs increased the total root surface of winter wheat by 95–341% and by up to 112–245% for barley.

Perhaps the main mycorrhizal-associated mechanism enhancing plant PAE is the increase of explored soil volume by the AM fungal hyphae, which can extend plant access from millimeters to centimeters from root surface. Fungal hyphae can also access soil pores that root hairs cannot due to their smaller diameter (20–50 um) (Figure 1B). Moreover, AM roots can improve water and nutrients uptake efficiency compared to non-colonized roots due to a lower C cost per unit of hyphal surface related to the root surface (Jansa et al., 2003a; Jakobsen et al., 2005; Gregory, 2006; Schnepf et al., 2008).

There is a complex interplay between root architecture and AM fungi and, as expected, root traits can influence how plants respond to mycorrhizal colonization (Newsham et al., 1995; Smith and Read, 2008). It is suggested that species with root systems characterized by low root hair length and density, and roots with relatively large diameters would display the greatest growth benefits from the symbiosis (Brundrett, 2002; Fitter, 2004; Smith and Read, 2008), especially under P-limiting conditions. Several studies have corroborated this assumption by making this comparison between wild and agricultural species, reporting associations between root traits and MGR (Baon et al., 1994a; Declerck et al., 1995; Schweiger et al., 1995; Jakobsen et al., 2005). However, a recent meta-analysis carried out by Maherali (2014) does not support this hypothesis.

Usually, root system architecture is also frequently modified before and following the establishment AM symbiosis (Scannerini et al., 2001; Hodge et al., 2009), especially through some fungal exudates, known as Myc-factors (Figure 1; Maillet et al., 2011; Mukherjee and Ané, 2011). These signal molecules are exuded even in the absence of a host plant and are involved not only in symbiotic signaling stimulating colonization, but also acting as plant growth regulators by modifying root development in some plant species (Maillet et al., 2011; Mukherjee and Ané, 2011). The formation of lateral roots has been found to be the most affected trait, making roots progressively more branched, probably to increase the number of suitable sites for colonization (Harrison, 2005). However, mycorrhizal-induced modifications on root traits are still poorly understood and seem to vary according to specific plant-fungal combinations, (Schellenbaum et al., 1991; Berta et al., 2005; Fusconi, 2014). In the case of wheat and barley, evidences are controversial as well. Behl et al. (2003) found a significant increase of total root length in wheat colonized by *G. fasciculatum*, being up to 90% higher than control plants when co-inoculated with *Azotobacter*. The same pattern was found by Al-Karaki and Al-Raddad (1997), who studied the response of two durum wheat genotypes to AM colonization, detecting an increase of 25 and 20% in root length. On the other hand, AM fungal inoculation decreased wheat root length and surface area under high rates of P application in a calcareous soil (Mohammad and Malkawi, 2004).

#### Organic acid anion and phosphatase exudation

It has been suggested that AM fungi may have biochemical and physiological capacities to increase plant PAE through the uptake of P from sparingly available forms in soil, being the exudation of protons, phosphatases and LMWOAAs the suggested mechanisms involved in these processes (Figure 1; Tarafdar and Marschner, 1994a; Koide and Kabir, 2000; Klugh and Cumming, 2007).

AM fungi possess many genes encoding acid phosphatases (EC 3.1.3.2, ACP) in their genomes, with at least seven genes expressed in *Rhizophagus clarus* (Sato et al., 2015). However, exudation of phosphatases was mostly associated with the cell wall (Olsson et al., 2002) and their presence in the rhizosphere has been demonstrated only in limited cases (Tarafdar and Marschner, 1994a; Koide and Kabir, 2000). The magnitude of these processes is questioned as it is difficult to isolate the effects of plants, fungi and others microorganisms present in the experiments under unsterile conditions (Joner and Jakobsen, 1995; Joner et al., 2000). However, Sato et al. (2015) in an experiment with separated compartments for hyphal growth, collected exudates from soil solution, sand culture and *in vitro* monoxenic culture, providing strong evidence that the corresponding acid phosphatase activity was originated from *R. clarus*. Little information is available about the relationship between AM symbiosis and changes in enzymatic exudation and activity patterns in wheat and barley. Rubio et al. (1990) found out a positive correlation between wheat colonization by AM fungi and acid phosphatase activity in roots and soil, mainly under P-limiting conditions. Using a different experimental approach with separated compartments for hyphal growth, Tarafdar and Marschner (1994a,b) observed depletion in organic P content with a concomitant increase of phosphatase activity when wheat was colonized by *Glomus mosseae* (Nicol & Gerd) Gerd & Trappe. The same trend was found for barley in a 10 years' field trial where P-deprived plants presented higher colonization by AM fungi and higher phosphatase activity than fertilized treatments (Goicoechea et al., 2004). In this sense, Ye et al. (2018) in a recent report show the importance of phosphatase activity in P acquisition by non-AM colonized barley efficient genotypes through direct changes of rhizosphere P fractions. Nevertheless, the interaction of AM association with the phosphatase activity and the subsequent P acquisition by efficient genotypes is still unclear.

The phosphate-solubilizing activities of AM fungi are still controversial although AM plants have generally been shown to increase the uptake of insoluble Pi (Yao et al., 2001; Tawaraya et al., 2006; Klugh-Stewart and Cumming, 2009). In many studies, mycorrhizal inoculants proved to alter the composition and/or amount of total LMWOAAs exuded by *Liriodendron tulipifera* and *Andropogon virginicus*, respectively (Figure 1; Klugh and Cumming, 2007; Klugh-Stewart and Cumming, 2009). However, direct evidence for solubilization of P by AM fungi has not been obtained so far. Despite that AM fungi might not exude LMWOAAs by themselves, they can, however, improve P solubilization and/or mineralization indirectly by stimulating the surrounding soil microbes via the exudation of labile C, thus increasing local nutrient availability in the hyphosphere and in soil patches beyond the root hairs (Hodge et al., 2010; Cheng et al., 2012; Jansa et al., 2013). Recently, Kaiser et al. (2015) using nanoscale secondary ion mass spectrometry imaging and ^13^C-phospho and neutral lipid fatty acids, traced the flow of recently photoassimilated C and found out that a significant and exclusive proportion of photosynthates was delivered through AM pathway and used by different microbial groups compared to C directly released by the roots.

The interaction between phosphate-solubilizing microorganisms with AM wheat and barley plants has been assessed by some researchers, with positive responses on growth and P uptake. Omar (1998) observed that the interaction between *Funneliformis constrictum* and the rock-phosphate-solubilizing *Aspergillus niger* and *Penicillium citrinum* fungi significantly increased biomass production of wheat plants under all experimental conditions tested. The effect was more evident in non-sterilized conditions. Bacteria from the *Azotobacter* and *Pseudomonas* genera also improved AM wheat growth under field and pot conditions, with positive correlation between AM colonization and *Azotobacter* survival in the rhizosphere (Kucey, 1987; Behl et al., 2003; Zaidi and Khan, 2005; Yousefi et al., 2011). Singh and Kapoor (1999) analyzed the effect of *Bacillus circulans, Cladosporium herbarum* and an isolated AM fungus in wheat where larger populations of phosphate-solubilizing microorganisms in the rhizosphere of mycorrhizal roots and an enhanced P acquisition in combined inoculation were found. Similarly, the inoculation with *Penicillium variable* alone negatively affected the biomass production of wheat. However, when applied in combination with *Azotobacter chroococcum, Pseudomonas striata* and the AM fungus *G. fasciculatum*, grain yield significantly increased compared with the other treatments (Zaidi and Khan, 2005).

In another study, wheat grain yield was enhanced by 92.8% in the presence of the rhizobacteria *Pseudomonas fluorescens* and *Burkholderia cepacia* and the AM fungus *Claroideoglomus etunicatum* (Saxena et al., 2013). The synergistic effect of combined inoculation with plant growth-promoting rhizobacteria and AM fungi on wheat was also proved to be effective under field conditions. It was shown that the combination of *A. chroococcum* and *Bacillus sp*. with *G. fasciculatum* significantly increased the dry matter by 2.6-fold and grain yield by 2-fold when compared to the control (Khan and Zaidi, 2007). In another field study, Mehrvarz et al. (2008) found that although bacterial inoculation alone achievied the maximum biological yield, its application combined with AM fungi produced grains with higher weight.

#### Changes in P transporters

In general, AM plants have two different pathways for P uptake from the soil (Figure 1C) with different P transporters involved in both of them. The direct P uptake is the plant endogenous pathway, which occurs via root epidermis and root hairs, while in the AM pathway the external hyphae is the responsible for acquiring P from the medium and transport to intracellular symbiotic interfaces where it finally goes to the plant (Grace et al., 2009; Smith et al., 2011). According to their function, plant transporters involved in the direct pathway are expressed mostly in the root apex and root hairs (Gordon-Weeks et al., 2003) and down-regulated in more mature regions. However, up-regulation of genes encoding phosphate transporters proved to have little influence on P acquisition. Rae et al. (2004) studying transgenic barley plants over-expressing a gene encoding for a phosphate transporter found no improvement on P uptake under any of the tested conditions, suggesting that post-transcriptional mechanisms could be involved affecting the activity of these transporters. AM transporters are less known due to their obligate biotrophic nature, coupled with the fact that they are multinuclear and heterocaryotic organisms (Sanders, 1999), which make the use of traditional genetic approaches difficult (Maldonado-Mendoza et al., 2001). These authors observed that the expression of a phosphate transporter gene from the extra-radical mycelium of *Rhizofagus intraradices* was regulated in response to P concentrations in the environment surrounding the extra-radical hyphae and that it was modulated by the overall phosphate status of the AM fungus rather than the host plant (Maldonado-Mendoza et al., 2001). Another important aspect of the AM pathway is the presence of AM-inducible plant P transporters, which are generally present at much higher levels in AM roots than other P transporters (Javot et al., 2007). These transporters are responsible for the exchange of P between the fungal hyphae and plant cell. They have been found in all AM plants investigated, regardless their growth response to colonization, and are mainly expressed in the colonized cortical cells, specifically in the arbusculated cells which is the place where the nutrient exchange takes place (Bucher, 2007; Javot et al., 2007). Genes encoding for AM-inducible transporters have been described in cereals and include the HvPHT1.11 and HvPHT1.8 for barley and TaPHT1.8, TaPHT1.11, TaPHT1.12, and TaPHT1.14 for wheat (Teng et al., 2017).

The two P pathways were believed to be additive in their contribution to plant nutrient uptake, and it was assumed that direct pathway made a constant contribution to the total P uptake, while the AM pathway participated as an extra contribution in plants with positive growth responses (Pearson and Jakobsen, 1993). However, further investigations proved that AM colonization could reduce the direct uptake pathway in some species (even in plants that respond positively to the symbiosis as in *Medicago truncatula*), and deactivate completely in others (Liu et al., 1998; Smith et al., 2004). Therefore, in order to not become P deficient AM pathway should compensate the reduced contribution of direct pathway (Smith et al., 2011). Recent studies using radioactive P isotopes has shown that AM pathway contributed significantly to total P uptake on wheat and barley. In this sense, Smith et al. (2015) clearly demonstrated that indigenous AM fungi contribute to wheat P uptake in 6.5–21% of total plant P in field conditions and 3–40% when grown in pots. However, mycorrhizal wheat plants acquired less P and produced less biomass when compared to their non-mycorrhizal counterpart (Li et al., 2006; Grace et al., 2009). It was suggested that negative growth responses could be generated by suppression of the direct pathway in these species, especially in the plants with very low colonization. Conversely, Grace et al. (2009) found out that the magnitude of the negative responses of barley was independent of contrasting colonization by two AM fungal species (*R. intraradices* and *F. geosporum*). In addition, the expression of P transporters belonging to direct pathway in barley was not affected by the symbiosis as expected. Again, this indicated that possible post-translational modifications of regulatory components could be involved in the plant response.

## AM functional diversity

It is a general consensus that there is little specificity between AM fungal and host plant species, and that AM plants can be colonized by several AM fungal species at the same time (Merryweather and Fitter, 1998; Jansa et al., 2003b; Smith et al., 2011). However, the existence of different colonization patterns could imply certain preferences for specific AM fungal species, functional groups or the co-evolution strategies between specific plant-fungus associations (Smith et al., 2009; Chagnon et al., 2013; López-García et al., 2017). For instance, Mao et al. (2014) showed that these preferences can exist even across wheat cultivars as they found a variation in AM fungal community composition, displaying a complex pattern of cultivar-AM fungal interaction under experimental field conditions. Despite of the projection of this work, the study of the AM fungal diversity associated to wheat and barley is overall scarce. Considering the wide distribution and economic importance of these two species, only 131 and five AM fungal sequences in MaarjAM database, the most complete sequence database of Glomeromycota (Öpik et al., 2010), are associated to wheat (*Triticum* sp.) and barley (*Hordeum* sp.) respectively, out of 5,296 sequences belonging to Poaceae in the database. The few studies covering molecular diversity in roots of wheat have shown differences between in community composition associated to wheat and N-fixing crops (Bainard et al., 2014; Higo et al., 2016). Communities associated to wheat have also been found to vary during the growing season and depend on P fluxes and degree of fertilization (Wu et al., 2011; Bainard et al., 2014; Qin et al., 2015). The diversity of AM fungal communities associated directly with roots of wheat is overall high, including members of different taxonomic families (e.g., Manoharan et al., 2017), but being predominatly associated with *Funneliformis* spp., in conventional cropping, and *Claroideoglomus* spp., in organically managed systems (Dai et al., 2014). In agreement, with this result, one of the few studies analyzing AM fungi in roots if barley, found that the abundance of *Funneliformis* spp. were associated with high levels of P in soil, meanwhile *Claroideoglomus* spp. with lower levels of P (Cruz-Paredes et al., 2017), but harboring a high phylogenetic diversity as well (Manoharan et al., 2017). N fertilization has been seen another affecting AM fungal community composition in barley and interacting with the plant-fungus P trade, as tends to decrease the efficiency in the interexchange (Williams et al., 2017).

The lack of information on molecular diversity has been in some manner compensated with morphological studies of spore communities. In this context, a high taxonomic diversity has been found. Aguilera et al. (2014, 2017) analyzing spore morphology on acidic soils under continuous wheat cropping, found 24 AM fungal species, being *Acaulospora* and *Scutellospora* the dominant genera. In another study under similar conditions in acidic soils, Castillo et al. (2016b) described 26 fungal species with a prevalence of *Acaulospora* and *Claroideoglomus*. This dominance of *Acaulospora* spores in soils cropped with wheat was also observed by Hu et al. (2015) in North China and by Nadji et al. (2017) in Algeria, however in the last study Glomeraceae species was also detected as highly abundant.

The mycorrhizal growth response of a single host plant species can differ across AM fungal species, and in the same way, colonization by the same AM fungal isolated can result in different growth responses in different plant species or genotypes (Feddermann et al., 2010; Smith et al., 2011; Castillo et al., 2016a). Indeed, previous studies have demonstrated a high variability in the symbiotic response of different combinations of host plant and AM fungi (e.g., Smith et al., 2004; Avio et al., 2006; Jansa et al., 2008). Variations in MGR have also been revealed across wheat cultivars, which can range from −2% to 107% in different genotypes (Azcón and Ocampo, 1981). On the other hand, Graham and Abbott (2000) showed a huge variation in MGR when testing several AM fungal isolates in symbiosis with wheat, being *Scutellospora calospora* the only one promoting higher plant biomass. In a study in wheat showed that MGR by different AM fungal species and their combination or with *F. mosseae* alone resulted in negative growth responses, while positive responses were reported when inoculated with *R. clarum* (Talukdar and Germida, 1994). This variability in mycorrhizal response comes from the fact that AM fungi are functionally diverse both inter- and intraspecifically (see for example Koch et al., 2004, 2017; Antunes et al., 2011). Differences among AM fungal species have been suggested to exist in the colonization rates in roots and soils depending on the AM fungal colonization pattern (Hart and Reader, 2002; Powell et al., 2009). Perhaps, although morphological traits seem to be well-conserved across AM fungal phylogeny, i.e. morphological traits into the same species and related clades are similar, most of variation in plant growth promotion and P uptake occurs indeed intraspecifically (Munkvold et al., 2004; Koch et al., 2017). In general, it had been assumed that morphological traits, such as the hyphal lenght in soil, could be good predictors of P uptake. However, the above mentioned results on huge variabilities in plant P uptake on morphological and phylogenetically similar fungal isolates redirects the question toward which fungal functional trait have to be measured to understand soil-plant P dynamics in agricultural systems. Therefore, functional diversity among AM fungal species and genotypes need to be considered.

## Future perspectives

Despite displaying negative responses in some studies and being considered as non-responsive by many authors, wheat and barley plants presented positive growth and P responses by performing AM symbiosis (Tables 1A,B respectively). There could be factors involved in this large PAE variation and the processes affecting both AM function and its benefits are still unknown. The question is complex due to the many factors are involved: plant genotype and fungal functional diversity, as well as their mutual compatibility, soil variable conditions or agricultural management needs to be studied. Indeed, the fact is that a major part of the research carried out in the interaction between crop cereals and AM fungi has only involved a handful of AM fungal isolates. In addition, there is little information available regarding the effect of different -or combined- AM fungal taxa colonization and different genotypes of wheat and barley on root morphology, development, exudation pattern, interaction with PGPR and/or P-solubilizing fungi, and the interplay between the two pathways of P uptake.

It is widely accepted that AM plants access to poorly available sources more effectively than non-colonized plants, but the mechanisms by which they are operating at field are not well understood (Smith et al., 2015). Studies using more than one crop cultivar and multiple AM species and genotypes should be carried out in order to analyze the effect of fungal diversity on PAE related traits as root length, root hair angles, changes on root-mycorrhiza exudation patterns and degree of inhibition (or not) of plant P transporters. In addition, these studies should be traced along different stages of development, until grain production, as it was found that although mycorrhization could hamper biomass production, it enhanced P acquisition and final grain production (Pellegrino et al., 2015). Isotopic, spectroscopic and molecular techniques coupled to new experimental designs could help identify some of the mechanisms mentioned above and the genetic background behind the different responses. In this sense, we suggest an inclusion of the Carbon costs related to all P acquisition traits (not only root architeture), specially those involved and altered by mycorrhizal colonization, in order to support accurate phenotyping for breeding programs focused on lowering P fertilizer inputs (Figure 2).

## Author contributions

PCa wrote the manuscript. FB was the main advisor and revisor of the document. PCo contributed on wheat and barley agronomical aspects and microorganisms effects on PAE. AS contributed to the construction of the document and the figures, and on all features related to phosphorus. JL-R provided insights on mycorrhizal signaling and the physiological effects upon colonization. AL-G also contributed on mycorrhizal aspects, highlighting the importance of functional diversity.

### Conflict of interest statement

The authors declare that the research was conducted in the absence of any commercial or financial relationships that could be construed as a potential conflict of interest.
